# Risks of spontaneously and IVF-conceived singleton and twin pregnancies differ, requiring reassessment of statistical premises favoring elective single embryo transfer (eSET)

**DOI:** 10.1186/s12958-016-0160-2

**Published:** 2016-05-03

**Authors:** Norbert Gleicher, Vitally A. Kushnir, David H. Barad

**Affiliations:** The Center for Human Reproduction, 21 East 69th Street, 10021 New York, N.Y. USA; The Foundation for Reproductive Medicine, New York, N.Y. USA; The Rockefeller University, New York, N.Y. USA; Department of Obstetrics and Gynecology, Wayne Forrest School of Medicine, Winston Salem, N.C. USA; Department of Obstetrics and Gynecology, Albert Einstein College of Medicine, Bronx, N.Y. USA

**Keywords:** Obstetrical outcomes, Singleton pregnancy, Twin pregnancy, Elective single embryo transfer

## Abstract

A published review of the literature by Dutch investigators in 2004 suggested significant outcome differences between spontaneously - and in vitro fertilization (IVF) - conceived singleton and twin pregnancies. Here we review whether later studies between 2004–2015 confirmed these findings. Though methodologies of here reviewed studies varied, and all were retrospective, they overall confirmed results of the 2004 review, and supported significant outcome variances between spontaneously- and IVF-conceived pregnancies: IVF singletons demonstrate significantly poorer and IVF twins significantly better perinatal outcomes than spontaneously conceived singletons and twins, with differences stable over time, and with overall obstetrical outcomes significantly improved. Exaggerations of severe IVF twin risks are likely in the 50 % range, while exaggerations of milder perinatal risks are approximately in 25 % range. Though elective single embryo transfers (eSET) have been confirmed to reduce pregnancy chances, they are, nevertheless, increasingly utilized. eSET, equally unquestionably, however, reduces twin pregnancies. Because twin pregnancies have been alleged to increase outcome risks in comparison to singleton pregnancies, here reported findings should affect the ongoing discussion whether increased twin risks are factual. With no risk excess, eSET significantly reduces IVF pregnancy chances without compensatory benefits and, therefore, is not advisable in IVF, unless patients do not wish to conceive twins or have medical contraindications to conceiving twins.

## Background

A groundbreaking study by Templeton and Morris in 1998 [1] ended with remarkable speed the worldwide glut of high order multiple births with in vitro fertilization (IVF). Improvements in implantation rates without concomitant changes in the clinical practice of transferring multiple embryos had been the cause of this glut. Templeton and Morris accomplished this remarkable feat by demonstrating that in younger, good-prognosis patients transfer of two embryos (2ET) sufficed to achieve excellent pregnancy rates, while avoiding high-order multiples from triplets onwards. Not less important was their concomitant observation that following 2ET, pregnancy success was in good-prognosis patients not diminished in comparison to transfers with more embryos.

The transition from multiple embryo transfer to 2ET, therefore, went smoothly and quickly because Templeton and Morris demonstrated that this practice change improved outcomes (i.e., significantly lowered the potentially risky high-order multiples) without incurring any detrimental outcome effects (pregnancy rates remained the same).

This at the time quite revolutionary step in the development of IVF, however, has to be differentiated from arguments in favor of elective single embryo transfer (eST), first proposed by Vilska et al. in 1999 [2]. Still dissatisfied by the high twin pregnancy rates with 2ET, these authors proposed that 2ET be replaced by eSET since only births of singleton offspring should be considered desirable IVF outcomes. Singleton pregnancies, of course, could only be “guaranteed” by broad utilization eSET.

Since eSET unquestionably, reduces pregnancy and delivery chances [3], in contrast to Templeton and Morris’ switch from multiple embryo transfer to 2ET, switching from 2ET to eSET even in good-prognosis patients came at considerable cost. For proponents of eSET such a price tag was warranted because twin births were supposedly associated with significantly higher complication rates than singletons, and, therefore, also increased costs to society [4].

The assumption that IVF-associated twin pregnancies are riskier and costlier for mothers and offspring (and society) than singleton deliveries, thus, represents the core of all advocacy in favor of eSET. Should this basic assumption be proven false, then there no longer exist medical indications for eSET since it clearly reduces pregnancy and live birth chances [3]. The only remaining indications for eSET then would be patients who wish to avoid twins or exhibit medical contraindications to conceiving twins.

Since our analyses of the literature suggested that in a prospective infertility treatment paradigm claims of significantly increased maternal and neonatal twin risks were statistically flawed, we have previously disagreed with the concept of eSET [5, 6].

Our reasoning is based on: (i) Correct statistical analyses require risk outcome comparisons for similar outcomes. As twin pregnancies produce two, and singleton pregnancies only one offspring, this most basic statistical rule is not met by comparing outcomes between one twin and one singleton pregnancy. While such a comparison is appropriate in a retrospective obstetrical paradigm, it is flawed in a prospective infertility paradigm, where patients and treating physicians are seeking best treatment for patients who are desirous of at least two more children. In such a paradigm, the correct statistical comparison is a comparison of one twin pregnancy to two consecutive singleton pregnancies, with both ultimately resulting in birth of two children.

We are aware of only three studies that investigated risk comparisons correctly. All other studies in the literature, therefore the vast majority on which eSET is based, utilized statistically inappropriate retrospective comparisons of one twin to one singleton pregnancy. Data of all three properly conducted studies were very similar, and no longer demonstrated significantly increased risk profiles for IVF twin pregnancies.

Of those three studies, however, only one recent Swedish study had been published by time of submission of this manuscript [7]. We had the opportunity to see the other two studies during the peer review process, and one of them has since also appeared electronically [8]. Both published studies demonstrated very similar results in still reporting mildly higher perinatal risks for twins than two consecutive singletons; but the recorded increased risks were clinically really irrelevant and compensated by some decreased risks in consecutive singleton pregnancies, especially in the Swedish study.

As previously noted, the authors of the Swedish study, unfortunately, misinterpreted their own results [7]. Correctly interpreted, the Swedish study also demonstrates no longer clinically significant increased risks for mothers and offspring in twin pregnancies. The only significant findings were higher cesarean section rates and a minimal increase in mild prematurity, with no reflection whatsoever in neonatal morbidity and mortality or even NCIU admissions. Remarkably, the study by La Sala et al. also demonstrated no increase in NCIU admissions but demonstrated significantly increased miscarriage rates with two consecutive singleton pregnancies [8].

Both of these studies, therefore, confirm what we concluded from reanalyzes of published data of other study formats [5, 6, 9] that, if outcome comparisons are correctly made in a prospective fertility paradigm, twin pregnancies in comparison to two singleton pregnancies, no longer appear to demonstrate clinically significant increased perinatal risks.

(ii) But in addressing this issue, a second statistical error in the medical literature also requires correction, and is the primary subject of here presented review: Studies claiming increased twin risks also assumed that maternal and neonatal outcomes of spontaneously conceived pregnancies and those conceived via IVF are the same. However, already in 2004, Helmerhorst et al. concluded after reviewing the literature that IVF-conceived twins experienced approximately 40 % lower perinatal outcome risks than spontaneously conceived twins, while IVF-conceived singletons actually demonstrated significantly increased perinatal risks in comparison to spontaneously conceived singletons [10]. These two opposing observations for singleton and twin pregnancies, therefore, suggested that, independently from above under (i) described statistical considerations, IVF twin risks in comparison to IVF singleton pregnancies are, likely, significantly overestimated for yet another reason [5, 6, 9].

We here, therefore, present a reviews of published data on this subject since Helmerhorst’s publication over a decade ago in order to determine whether those findings have been holding up under more current practice.

## Sources

We systematically reviewed the English literature between 2004 and 2015 for articles that addressed outcome comparisons between spontaneously conceived and IVF-conceived singleton pregnancies and/or twin pregnancies. Searches were conducted under appropriate key words via PubMed, Medline and the Cochran Library. We also checked Clinical.Trials.gov for ongoing studies on the subject. Based on suggestions during the review process, we also searched Popline, EMBASE and Web Science but were unable to discover additional relevant publications in English.

To preclude outcome biases from single center practices, we concentrated the search on systemic reviews and on publications using multicenter and/or national data sets, and excluded publications that reported only single center experiences involving only small patient samples. The principal reason for this decision was that we did not want outcome comparisons to be potentially biased by obstetrical/neonatal practice patterns, which do not represent widely practiced standard of care. Considering widely differing study designs in all published studies, which did not allow for a meta-analysis of published data, our statisticians advised us to avoid small, single center studies that could introduce single center practice biases.

Since keywords could not reflect the here reviewed subject in isolation, we conducted our data base search with the following phrases: <outcome comparisons/cost comparisons/maternal outcome comparisons/perinatal outcome comparisons/neonatal outcome comparisons between spontaneous and IVF/ART pregnancies > .

**Fig. 1:**
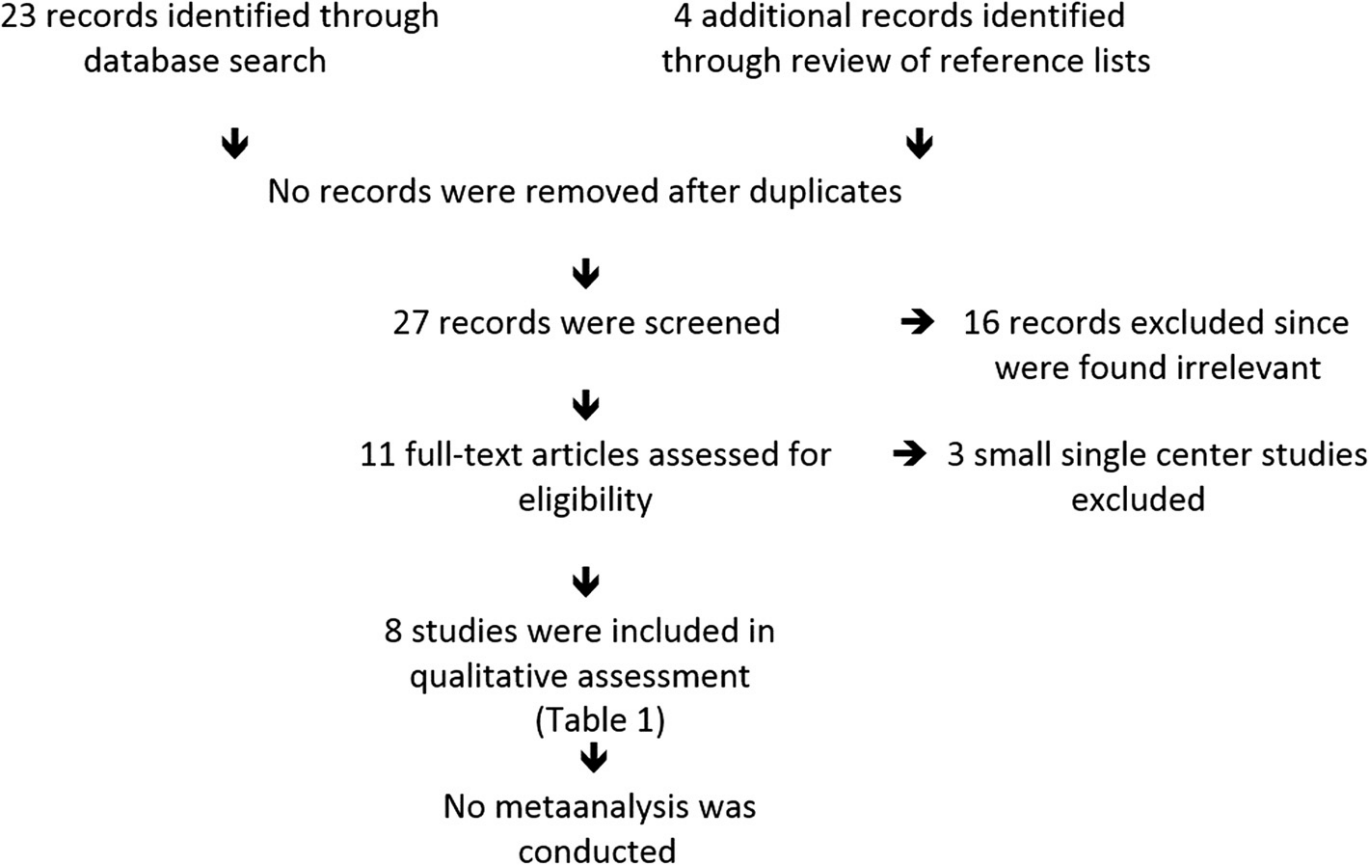
Flow of information according to PRISMA guidelines

Figure 1 summarizes the flow of information.

## Study selection

We identified 23 records through database searches, added an additional 4 via reference list searches and an additional during the review process for a total of 28 records, which were screened by one of the authors (N.G.) Sixteen were identified as irrelevant to here discussed subject. The remaining 12 articles were assessed for eligibility by all three authors, among those a relatively recent publication from Sweden [7], which we recently reviewed elsewhere in detail [9].

Three studies were disqualified from further evaluation because they reported small data sets from single centers, where center-specific clinical practice patterns could have influenced outcomes. This study, therefore presents data from 8 publications, all either large multicenter, national or multinational data sets. Table 1 summarizes them in order of historical appearance in the literature.

**Table 1:** Comparisons of obstetrical outcomes between spontaneously conceived and IVF pregnancies

Authors	Year	Study format	Singletons	Twins	Comments
Källén et al [11]	2010	National		X	Significant increase in IVF of PTB (<32 weeks); No difference in LBW
Pinborg et al [15]	2013	Review	AOR 1.27, (95 % CI 1.08, 1,49)		Even in same mother an IVF offspring has more PTB than non-IVF Offspring
Sazonova et al [7]	2013	This study is only indirectly relevant to here reviewed subject but is listed because it is the only study, which correctly compared in a large national population outcomes of twin pregnancies in comparison to *two* consecutive singleton pregnancies. Unfortunately, as previously in detail reviewed by us, the authors misrepresented their data in discussing their conclusions [9]. A correct analysis of their data demonstrated no clinically significant outcome differences in either maternal or neonatal outcomes, with AORs listed in the reference. The study, however, did *not* comment on differences between spontaneously- and IVF-conceived pregnancies.			
Anbazahagan et al [12]	2014	MCPT	no significant difference	X	No difference between IVF and spont. twins but small size and prospective study
Henningsen et al [16]	2014	Scandinavian population study	AOR 1.54 (95 % CI 1.28, 1.85)	X	IVF singletons had increased neonatal death risk. IVF twins had lower risk, which was lost when restricted to opposite-sex twins
Dar et al [13]	2014	Review and meta-analysis; Study does not comment on differences In outcomes between spontaneously and IVF-conceived singletons and twins but demonstrates significantly increased PTB risk for blastocyststage embryo transfer in comparison to cleavage-stage embryo transfer, a finding with relevance to here discussed topic since blastocyst-stage embryo transfer is a prerequisite for eSET.			
Declercq et al [17]	2015	Cohort	AOR for PTB 1.23AOR for LBW 1.26		Both AORs are in comparison to a subfertile patient group: Risks of singletons among IVF patients and in a sub-fertile patient group were, both, higher than in normally fertile population.
				AOR for PD 0.55 in comparison to fertile controls	
				AOR for PF 0.15 in comparison to subfertile controls	
Henningsen et al [14]	2015	Cross-border Scandinavian cohort study demonstrating significant declines over last 20 years in stillbirth and infant deaths for IVF singletons and twin deliveries, with “fewer” IVF twins being stiiborn or died during year 1 of life compared to spontaneously conceived twins (presumably due to fewer monozygotic twins among IVF twins). In addition IVF singletons demonstrated a significant decline in being born preterm and very preterm.			

*AOR* adjusted odds ratio; *PTB* preterm birth; *X* – in comparison to; *MCPT* multicenter prospective trial, *LBW* low birth weight; *PD,* perinatal death

Because methodologies and study end points greatly varied, a metaanalysis of reported data was not possible. The table, however, attempts to summarize individual study outcomes in their relevance.

## Results

The first study addressing the issue after Helmerhorst’s review of the literature [10] was by the Swedish group of Källén et al. [11]. Using national health registries in Sweden for the period of 1982–2007, they compared 1545 pairs of different-sex (dizygotic) twins born after IVF with 8675 non-IVF dizygotic twin pairs. Main outcomes were length of gestation, birth weight, respiratory complications and jaundice. As the only statistically significant finding, they reported in IVF-twins increased risk for preterm delivery before 32 weeks gestational age.

These results not only contradict the data accumulated by Helmerhorst et al. [10], but also data of all other later studies published on this subject, listed in Table 1. They are also contradicted by this study’s own finding of no difference in low birth weights. It is difficult to understand how prematurity under 32 weeks can be more prevalent; yet, birth weights are not.

The authors, themselves, indeed, raised doubts about the reliability of their data sets, and the length of time (25 years) in which these outcomes accumulated. Such a time span, of course, involves significant practice changes.

Among subsequently performed studies, only the study by Anbazhagan et al. demonstrated no differences between IVF-conceived and spontaneously conceived pregnancies [12]. All other studies, indeed, did demonstrate statistically highly significant differences between IVF-associated and spontaneously conceived gestations; with IVF-singletons uniformly doing much poorer than spontaneously conceived singletons, and IVF-conceived twins doing much better than spontaneously conceived twins (Table 1).

The Table also demonstrates, based on the studies by Dar et al. [13] and Henningsen et al., [14] that, as one would expect, perinatal/neonatal outcomes have not remained static over time but have been improving for singletons as well as twin deliveries. This observation, alone, reemphasizes the need for utilization of contemporary risk outcome data when comparing outcome risks between singleton and twin pregnancies.

As the table also demonstrates, adjusted odds ratios (AORs) vary, depending on which outcomes are assessed; but studies by Pinborg et al. [15], Hennigsen et al. [14, 16] and Declercq et al. [17] do suggest that obstetrical outcome studies in spontaneously conceived pregnancies vary from IVF-conceived pregnancies approximately by a combined AOR of 0.25–0.50, with IVF twins doing much better and IVF singletons much poorer. These results also suggest that overestimations of twin risks increase with severity of risk. In other words, risk exaggeration for perinatal/neonatal death carries an AOR of approximately 0.50 (i.e., appears overestimated by ca. 50 % for IVF twins), while milder risks, such as preterm birth or low birth weight carry overestimates of AOR of only approximately 0.25 (i.e., 25 %). These results, thus, fully confirm the earlier analysis of the literature by Helmerhorst et al. in 2004 [10] and, in addition, add significant further detail.

The study by Declercq et al. adds further subtlety because it for the first time also presents data on a sub-fertile (i.e., infertile patient population receiving treatments other than IVF) non-IVF control group. It demonstrates that this additional patient group falls in respective risk in-between spontaneously conceived and IVF-conceived pregnancies (Table 1) [17]. This observation, therefore, further validates prior and more recent observations of divergent risk profiles for singleton and twin pregnancies, depending on mode of conception.

## Discussion

Here presented review confirms the significant divergence of risks for IVF- and spontaneously conceived singleton and twin pregnancies, already in 2004 reported by Helmerhorst et al. [10]. Moreover, this review suggests that the risk divergence, reported by Helmerhorst et al., despite generally improving perinatal and neonatal outcomes for singleton as well as twin pregnancies has been approximately maintained.

As this study also revealed, recently published papers on this subject suffer from the same shortcomings Helmerhorst et al. decried when, first time, investigating this subject over 10 years ago [10]. Studying this subject by metaanalysis was, therefore, then and now impossible, though the consistency of findings between both studies is quite remarkable.

Since published perinatal/neonatal risk assessments of singletons and twins almost uniformly have been obtained from spontaneously conceiving obstetrical populations, they, therefore, indisputably exaggerate IVF-twin risks and underestimate IVF-singleton risks. At least for the most severe risks, like perinatal/neonatal deaths, risk exaggerations may be as high as 50 %, while milder risks may carry only biases of ca. 25 %.

None of the reviewed studies offer robust evidence for the reasons of observed discrepancies between spontaneous – and IVF-conceived pregnancies, and one is, therefore, left with only hypotheses: IVF twin pregnancies may demonstrate better outcomes since these pregnancies are recognized earlier than spontaneously conceived twins and, therefore, very likely receive more “tender loving care” at early stages of pregnancy. Early diagnosis of twin pregnancy may offer disproportionally more benefits than early diagnosis of singletons, since twin pregnancy, of course, carry significantly higher early pregnancy risks.

Here reaffirmed evidence of significant outcome differences between spontaneously – and IVF – conceived pregnancies raises additional doubts that IVF-associated twin pregnancies, indeed, increase perinatal risks in comparison to IVF-associated singleton births. If one then adds that historical comparisons of twin to single birth also overestimate twin risks by almost) 50 % (one study paradoxically claimed that second consecutive singleton deliveries demonstrate lower outcome risks than first singleton deliveries [7]), one is at best left with significant doubts and, likely, with a degree of conviction that alleged higher IVF-twin risks really may not exist.

Increased IVF-twin risks, however, represent the primary rational for the concept of eSET. In absence of an alleged increased risk associated with IVF twins, there is absolutely no reason for eSET because the literature undisputedly demonstrates that eSET reduces pregnancy chances in comparison to the transfer of two embryos [18]. The study of La Sala et al. again confirmed this fact in a single twin to two consecutive singleton pregnancy comparison [8].

Our review revealed yet another paradox in the current clinical utilization of eSET: The concept of embryo selection, as currently practiced, is, of course, closely related to the concept of eSET via attempts of selecting best embryos by blastocyst-stage culture and subsequent eSET at blastocyst stage (days 5/6 after fertilization). Dar et al., following a systematic review of the literature, recently, however, reported that blastocyst stage embryo transfer significantly increases the risk of preterm births for singleton IVF pregnancies in comparison to earlier (day 3) cleavage stage embryo transfers (AOR 1.32) [13]. In other words, embryo selection by blastocyst stage culture, primarily performed to select “best” embryos for eSET, actually significantly increases perinatal risks for singleton IVF pregnancies, thus providing further support for the argument that twin IVF pregnancy risks are significantly overestimated in comparison to IVF singleton risks.

The principal limitation of this study is absence of prospective studies, and of studies that correctly compare outcomes in IVF patients leading to births of two children between one twin and two consecutive singleton pregnancies. Since such studies are not available (and, likely, will not become available in the foreseeable future), clinical conclusions have to rely on individual study analyses, as here presented.

A better understanding of advantages and disadvantages of eSET, in our opinion, is of essence, as national IVF outcomes all over the world are affected by increasing utilization of eSET. While these trends, unquestionably, reduce twin pregnancies, they also to significant degrees appear to reduce regional IVF pregnancy rates in many parts of the world [19]. If reduction in twin pregnancies is a worthwhile compensatory expense for decreasing pregnancy and live birth rates, then further promotion of eSET policies would appear warranted. If, however, as by this study suggested, twin pregnancies do not produce added perinatal risk, then the significant decline in clinical pregnancy rates and an almost 40 % decline in live births in the Canadian province of Québec following introduction of a provincial eSET mandate [20], would have to considered poor public health policy, and propagation of eSET policies by professional societies, insurance companies and government agencies should be abandoned.

## Conclusions

Here presented data raise serious questions about the rapidly expanding IVF practice of prolonged embryo culture to blastocyst stage, followed by eSET. Since it is undisputed that eSET reduces clinical pregnancy chances in IVF when compared to two-embryo transfers [8, 18], proponents of eSET consider such reductions in pregnancy potential appropriately compensated by decreased maternal and especially neonatal risks from avoided twin pregnancies. In absence of increased risks from twin pregnancies, patient would, however, be only left with a deficit in pregnancy chances, and without compensatory benefits of any kind. Here presented review, therefore, adds significant doubts about the medical and economic validity of eSET.

The concept of eSET, therefore, requires serious reconsideration, unless patients want only one child to complete their family or have medical contraindications to twin pregnancies. In all other cases, eSET, as currently increasingly considered standard of care, actually, likely, harms pregnancy chances of infertile patients undergoing IVF cycles, therefore unnecessarily prolonging their time to pregnancy and increasing their medical costs.

### Details of ethics approval

As a review article of the literature no ethics approval was required.
